# Plant response to butterfly eggs: inducibility, severity and success of egg-killing leaf necrosis depends on plant genotype and egg clustering

**DOI:** 10.1038/s41598-017-06704-z

**Published:** 2017-08-04

**Authors:** Eddie Griese, Marcel Dicke, Monika Hilker, Nina E. Fatouros

**Affiliations:** 1 Laboratory of Entomology, Wageningen University, Droevendaalsesteeg 1, 6708 PB Wageningen, The Netherlands; 2 Institute of Biology, Freie Universität Berlin, Berlin, Germany; 3 Biosystematics group, Wageningen University, Droevendaalsesteeg 1, 6708 PB Wageningen, The Netherlands

## Abstract

Plants employ various defences killing the insect attacker in an early stage. Oviposition by cabbage white butterflies (*Pieris* spp.) on brassicaceous plants, including *Brassica nigra*, induces a hypersensitive response (HR) - like leaf necrosis promoting desiccation of eggs. To gain a deeper insight into the arms race between butterflies and plants, we conducted field and greenhouse experiments using different *B*. *nigra* genotypes. We investigated variation in HR and consequent survival of *P*. *brassicae* egg clusters. Impact of egg density, distribution type and humidity on HR formation and egg survival was tested. HR differed among plant genotypes as well as plant individuals. Egg density per plant did not affect HR formation. Remarkably, egg survival did not depend on the formation of HR, unless butterflies were forced to lay single eggs. Larval hatching success from single eggs was lower on plants expressing HR. This may be due to increased vulnerability of single eggs to low humidity conditions at necrotic leaf sites. We conclude that effectiveness of HR-like necrosis in *B*. *nigra* varies with plant genotype, plant individual and the type of egg laying behaviour (singly or clustered). By clustering eggs, cabbage white butterflies can escape the egg-killing, direct plant defence trait.

## Introduction

Plant defences against herbivory largely exhibit phenotypic plasticity, which is a powerful means to cope with threats because a target with variable traits is less likely to be hit^1^. Plants are well known to defend themselves against herbivorous insects by a wide range of strategies, some of which are genotypically fixed and employed constitutively, and numerous others are based on phenotypic changes that are induced in response to herbivore attack^2–5^. The range of phenotypic changes of a plant is determined by its genotype and its environment^6^. Hence, different accessions of a plant species exposed to variable environmental conditions may display variability with respect to the inducibility of their defensive responses.

Egg deposition by insects onto plants often represents the first step of infestation. Plant defences induced by insect oviposition may target the eggs themselves or the herbivorous larvae. Oviposition can inform plants about impending herbivory, and the “warned” plants prepare their defences against hatching larvae. Various plant responses are known to directly kill the eggs by ovicidal compounds, growth of plant tissue around the eggs, or the formation of neoplasms or necrotic leaf tissue (reviewed by refs 7-9). The formation of necrotic leaf tissue in response to insect egg deposition leads to detachment of eggs from leaves or desiccation of eggs and has been described as a hypersensitive-like response (HR)^10–16^.

From the insect’s perspective, the oviposition behaviour of a mother is an important determinant of successful reproduction^17–19^. Females invest in survival of their progeny by e.g. hiding the eggs, protecting them with sticky secretions that prevent parasitism, endowing them with predator-deterring compounds or by increasing egg size^8, 20, 21^. In herbivorous insects, clustering of eggs has been suggested as an adaptation to plant defences^8, 21, 22^.

Our understanding on the ecology and mechanisms of oviposition-induced plant responses is largely increasing^8, 13, 23–25^. However, some aspects of plant – insect egg interactions have remained largely unexplored such as (i) the ability of an herbivorous insect to counteract oviposition-induced plant responses, and (ii) the genotypic and individual variation of plant responses to insect oviposition. In contrast to these gaps in knowledge about plant – insect egg interactions, solid information is available on the variability of plant responses to feeding arthropods. Genotype-based plasticity of feeding-inducible plant defensive responses is well documented^5, 26–30^ and has been suggested to be shaped by the costs of this plasticity^31^. Furthermore, individual variation in the composition of plant defence-eliciting compounds that feeding larvae introduce into plant wounds is well known^32^, and some means of counteractions of larvae against feeding-induced plant defences have been detected^33–39^.

Here, we address the above-mentioned knowledge gaps in plant – insect egg interactions by studying the response of various accessions of the black mustard *Brassica nigra* to egg deposition by the large cabbage white butterfly (*Pieris brassicae*). The annual *B*. *nigra* shows diverse responses to eggs of *Pieris* spp., including accelerated flower and seed production as reproductive escape strategy^4, 40^, attraction of egg- and larval parasitoids^14, 40–43^, phenotypic changes that affect subsequent herbivore and parasitoid preferences and performances^15, 24, 43^, and HR - like necrosis^10, 14, 15, 41^. Different populations of *B*. *nigra* respond at different frequencies to singly laid *P*. *rapae* eggs or to clustered *P*. *brassicae* eggs by HR - like necrosis^14, 41^. Under both greenhouse and natural conditions, the hatching success of larvae from solitary *P*. *rapae* eggs was reduced when the plant expressed HR^14^.

For the gregarious *P*. *brassicae* which lays its eggs in clusters it is unknown as yet whether formation of egg-induced HR-like symptoms and the hatching success depend on the number of eggs per cluster. Although effects of HR-like necrosis on *P*. *brassicae* egg survival were previously studied under greenhouse conditions^41^, a thorough study examining the relationship between HR and survival of clustered eggs of *P*. *brassicae* under natural conditions is still lacking. To date, hardly anything is known about the ecological determinants of egg responses induced by herbivore oviposition. As shown in previous studies, egg load^45^ and abiotic factors such as temperature or water st﻿ress^10, 12, 46^ can negatively or positively affect egg-induced plant defences.

In the present study, we investigated the following specific questions by conducting field, greenhouse, and laboratory experiments: Does formation of HR-like symptoms by *B*. *nigra* in response to *P*. *brassicae* egg deposition differ (1) among plant accessions and (2) among individuals of a *B*. *nigra* accession? (3) Is mortality of clustered *P*. *brassicae* eggs dependent on the occurrence of HR and correlated with HR severity? (4) Does clustering of *P*. *brassicae* eggs counteract the plant´s HR? Since abiotic environmental conditions may significantly impact on phenotypic plant traits, we also investigated how (5) abiotic parameters (temperature, humidity, total radiation and sum of rain) affect the expression of the plant’s HR and mortality of butterfly eggs.

## Results

### Variation in HR among plant accessions: field data

**Figure 1:**
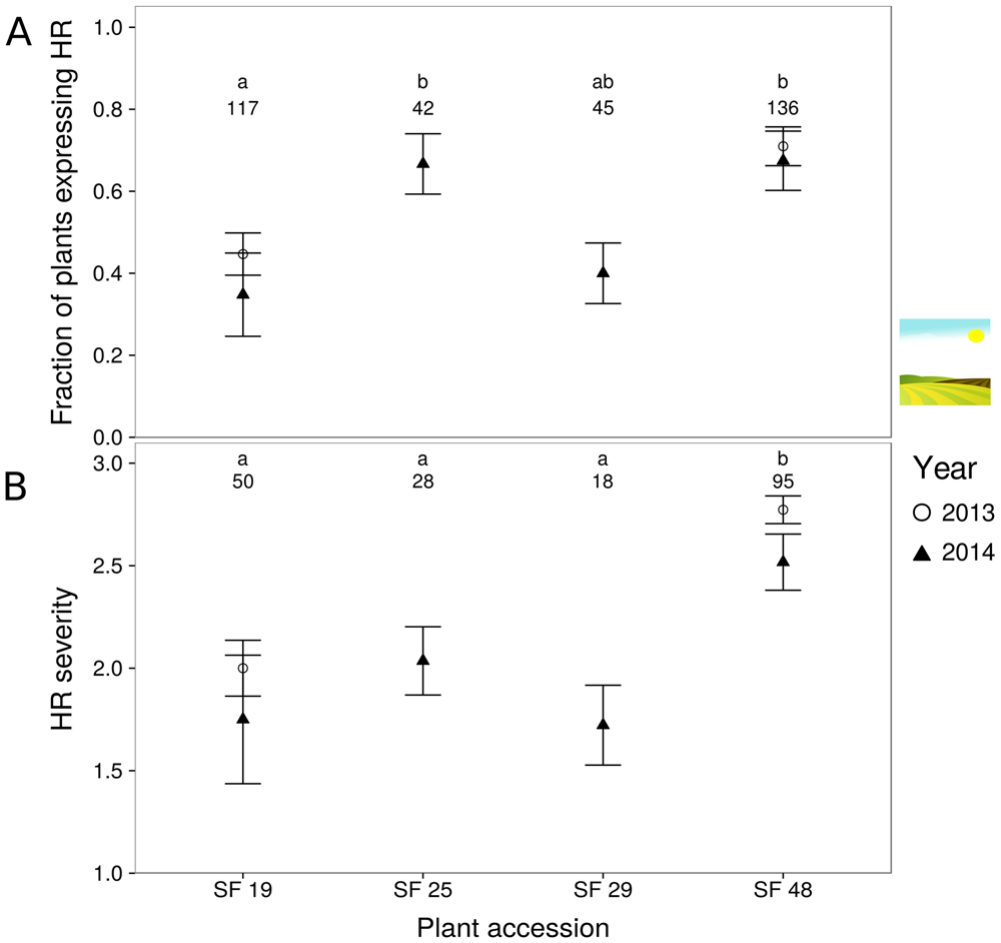
HR induced by *P*. *brassciae* eggs in *B*. *nigra* plant accessions used in common garden experiments in 2013 (only SF19 and SF48) and 2014. (**A**) Fraction of plants expressing HR (mean ± SE) per plant accession. (**B**) HR severity (mean ± SE) per plant accession (severity 1–3, compare Fig. S1). Numbers of plants tested for each accession are indicated above each data point, different letters indicate significant differences (*P* < 0.01, LHT post hoc test).

We used a common-garden setting to investigate whether four self-fertilised accessions of *B*. *nigra* show differences in expression of HR induced by *P*. *brassicae* eggs. Expression of HR was significantly influenced by the plant accession receiving the eggs (GLM, χ² = 24.74, df = 3, *P* < 0.001, Fig. 1A). Accessions SF25 and SF48 had a higher proportion of plants expressing HR than accession SF19. Moreover, HR severity was also significantly affected by plant accession (GLM, χ² = 69.32, df = 9, *P* < 0.001, Fig. 1B). HR severity of SF48 was significantly higher than HR severity of the three other plant accessions (Fig. 1B). We conducted this experiment in two consecutive years. The year of the experiment did neither influence the fraction of plants expressing HR, nor the HR severity for SF19 and SF48, which were tested in both years (GLM, χ² = 0.75, df = 1, *P* = 0.39 and GLM, χ² = 2.06, df = 1, *P* = 0.15, respectively, Fig. 1). In conclusion, some genotypes show a greater inducibility and severity of HR-like symptoms by *P*. *brassicae* eggs than others.

### Variation in HR among plant accessions and plant individuals: greenhouse data

In a greenhouse experiment we investigated the variation in expression of HR with respect to plant accession by examining 38 plant individuals belonging to two genotypes which were exposed to eight to ten butterflies that each laid five eggs.

#### Variation among plant accessions

Unlike under field conditions, neither the presence/absence of HR symptoms nor HR severity were significantly different between the two plant accessions SF19 and SF48 (GLM: χ² = 0.19, df = 1, *P* = 0.66 and χ² = 2.94, df = 2, *P* = 0.23).

#### Variation among individual plants

**Figure 2:**
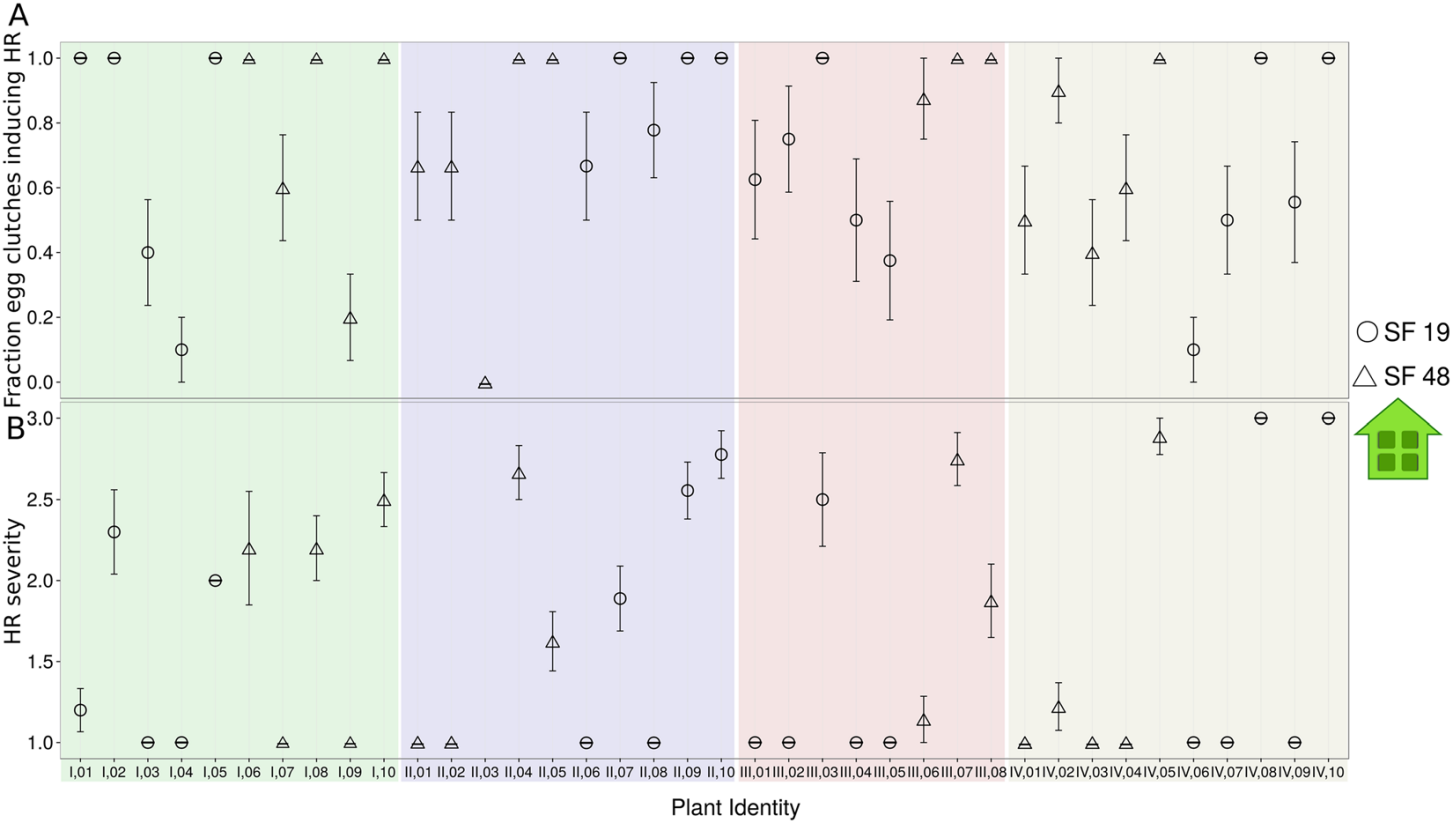
HR induced by *P*. *brassicae* eggs in different plant individuals from two accessions under greenhouse conditions. (**A**) Fraction of egg clutches expressing HR per plant (mean ± SD). (**B**) Severity of HR when HR is expressed in plants (mean ± SD). Four different replicates and therefore plant/butterfly sets were used and marked with differently shaded background and (Latin) numbers for plant identity. Both plant accessions, SF19 and SF48 are separated using different shapes for data points, (see explanation in the figure).

Both the presence of HR symptoms in response to individual egg clutches and HR severity varied significantly among plant individuals (GLM: χ² = 196.59, df = 37, *P* < 0.001 and χ² = 352.27, df = 74, *P* < 0.001). As seen in Fig. 2A except for one plant (II.03), all plants expressed HR at least for some of the egg clutches. Seventeen plants expressed HR in response to all egg clutches laid onto them. With respect to the severity of the expressed HR about half of the plants (N = 18) expressed the lowest HR severity in response to all deposited egg clusters, one plant always the medium severity and two always the strongest. All other plants expressed varying degrees of HR severity for the egg clutches laid onto them (Fig. 2B). In conclusion, the data suggest that HR severity (and inducibility) is strongly linked to the individual plant, and differs only slightly between different egg clutches on the same plant.

### Effects of HR on survival of clustered eggs: field and greenhouse data

*Brassica nigra* plants were infested with egg clutches of *P*. *brassicae* females either in the greenhouse or outside in a field experiment.

**Figure 3:**
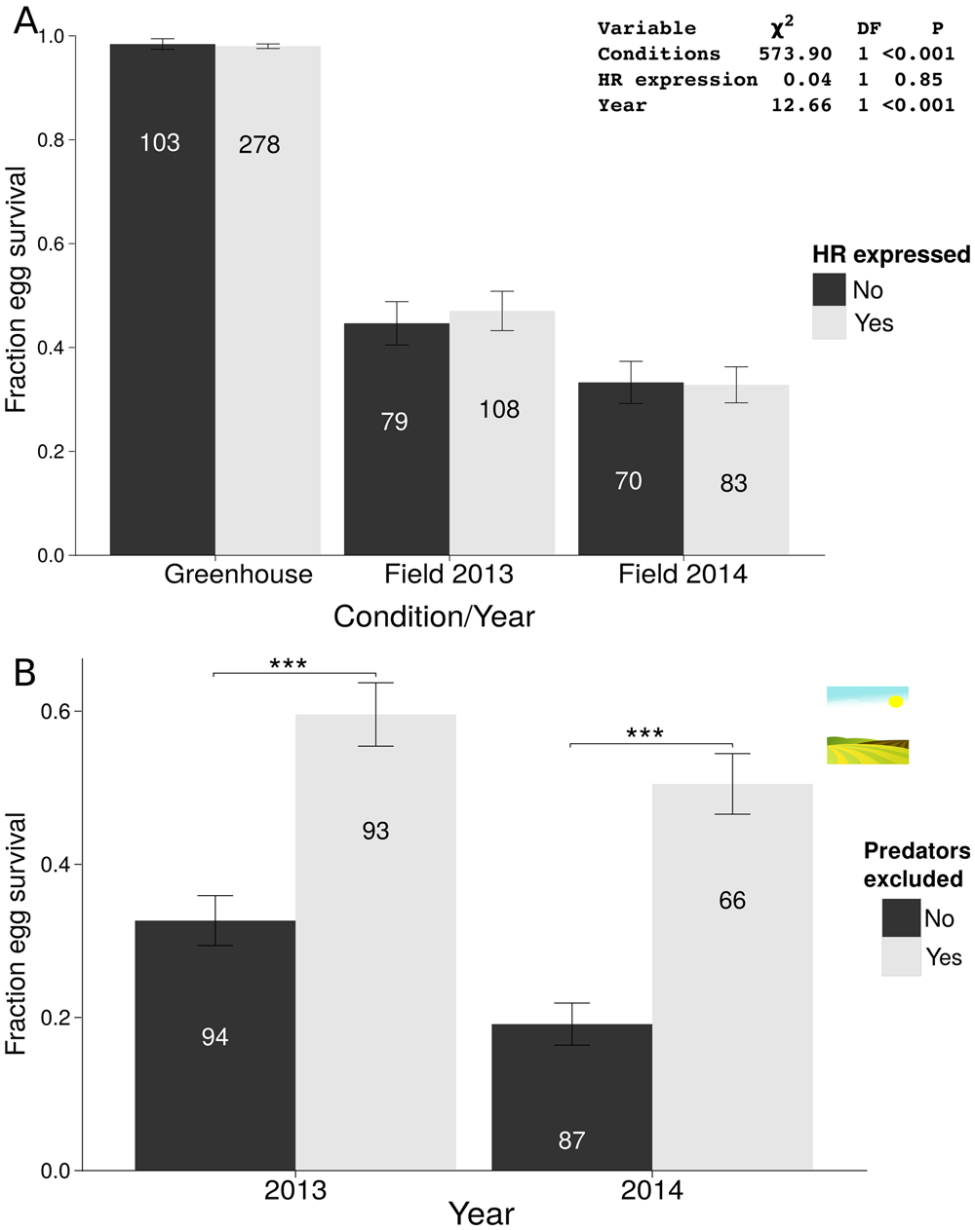
Effect of HR expression on survival of clustered *P*. *brassicae* eggs (mean ± SE) laid on *B*. *nigra* plants under greenhouse and field conditions (for 2013 and 2014). (**A**) The numbers within each bar represent the numbers of tested plants. The differences in egg survival until larval hatching between HR expressing (light grey) and non-HR expressing plants (dark grey) are not significant, GLM, family = quasibinomial. Differences between greenhouse and field conditions and between both years of field experiments are indicated by asterisks above the horizontal lines, GLM, family = quasibinomial. (**B**) Effect of predator/parasitoid exclusion on survival of clustered *P*. *brassicae* eggs (mean fraction ± SE) laid on *B*. *nigra* plants in the field. Plants were either covered with a fine mesh during the egg phase (predators excluded – light grey) or left uncovered (predators included – dark grey).

Survival of egg clutches did not depend on the expression of HR (GLM, χ² = 0.19, df = 1, *P* = 0.66), neither under greenhouse nor field conditions (GLM, χ² = 0.17, df = 1, *P* = 0.67 and GLM, χ² = 0.01, df = 1, *P* = 0.93, respectively, Fig. 3). Yet, egg survival was significantly greater in the greenhouse than in the field (GLM, χ² = 593.34, df = 1, *P* < 0.001). Moreover, under field conditions, survival rates were significantly higher in 2013 than in 2014 (GLM, χ² = 10.72, df = 1, *P* = 0.001). The large difference in egg survival rates between the greenhouse (almost 100% survival) and field (between 35 and 50% survival) could be due to abiotic factors as well as biotic factors, such as egg predation/parasitism. When egg clutches were protected by fine meshed nets from parasitoids and predators in the field, egg survival indeed significantly increased (from 33% to 60% in 2013, and 19% to 51% in 2014) in both years (MWU, 2013: W = 2700.5, *P* < 0.001 and 2014: W = 1256, *P* < 0.001, respectively, Fig. 3B. This suggests that HR is not an effective defence trait against clustered *P*. *brassicae* eggs.

### Effects of HR on survival of single versus clustered eggs

We investigated formation of HR in dependence of the egg clutch size (number of eggs) per plant and recorded egg survival in dependence of whether eggs were laid singly or clustered. Singly laid eggs were experimentally achieved by manipulating the female´s oviposition mode.

**Figure 4:**
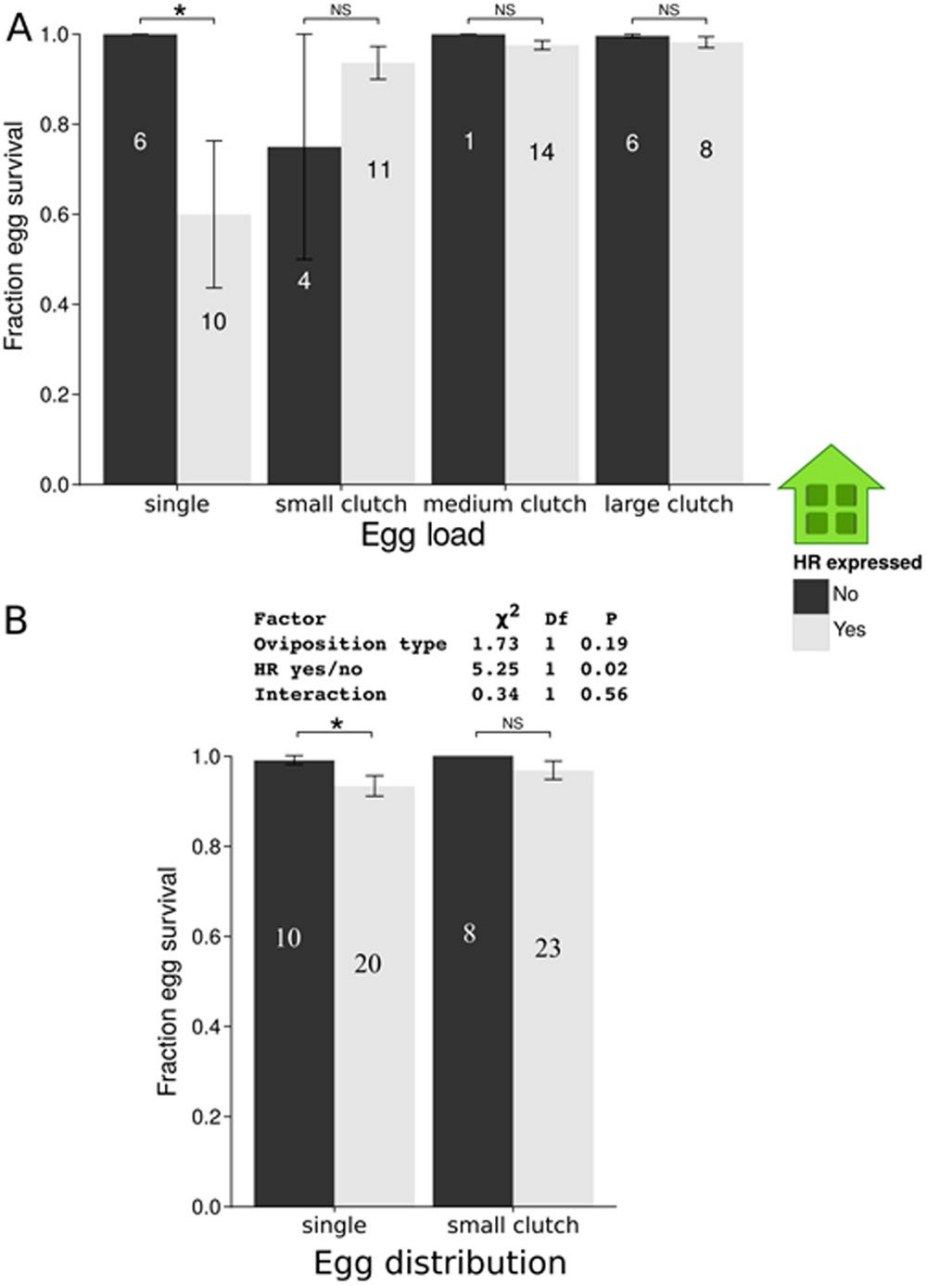
Fraction of *P*. *brassicae* egg survival under greenhouse conditions. (**A**) Impact of number of eggs per plant and egg distribution. Effect of HR expression on survival (mean fraction ± SE) of single *P*. *brassicae* eggs per plant and *P*. *brassicae* egg clusters of different size as stated on the X-axis (small: 10, medium: 50 and large: 90 eggs) laid on *B*. *nigra* plants. Survival rates of *P*. *brassicae* eggs on *B*. *nigra* plants expressing HR (light grey columns) or not (dark grey columns). Numbers in the columns represent the numbers of tested plants. (**B**) Impact of egg distribution. Effect of HR expression on survival (mean fraction ± SE) of 10 singly laid *P*. *brassicae* eggs and of a *P*. *brassicae* egg cluster (containing 10 eggs) on *B*. *nigra* plants under greenhouse conditions. Survival rates of eggs on *B*. *nigra* plants expressing HR (light grey columns) or not (dark grey columns). Numbers in the columns represent the numbers of tested plants. Significant differences are indicated using asterisks. *P < 0.05, ns: not significant, GLM.

Under greenhouse conditions, HR negatively affected survival of a single egg per plant (GLM, χ² = 6.35, df = 1, *P* = 0.01, Fig. 4A). However, when more eggs were laid per plant and when these were laid in small (N = 10) or larger groups (N = 50 or 90), the expression of HR did not affect egg survival (GLM, small clutch size/number of eggs: χ² = 1.80, df = 1, *P* = 0.18, medium: χ² = 0.83, df = 1, *P* = 0.36, and large: χ² = 1.40, df = 1, *P* = 0.24, Fig. 4A), while HR negatively affected survival when a female was forced to lay a single egg (GLM, χ² = 6.35, df = 1, *P* = 0.01, Fig. 4A).

In a second experiment, we focused on the question how HR affects egg survival if the number of eggs is the same per plant (here 10 eggs per plant), but the type of egg distribution on a plant differs (singly laid eggs *vs* clustered eggs). Egg survival was significantly lower on plants expressing HR than on plants that did not express HR (GLM, χ² = 5.25, df = 1, *P* = 0.02, Fig. 4B). The egg distribution (10 singly laid eggs vs. 10 eggs laid in a cluster) and the interaction between HR expression and egg distribution did not affect egg survival (GLM, χ² = 1.73, df = 1, *P* = 0.19 and χ² = 0.34, df = 1, *P* = 0.56, Fig. 4B). However, when the effect of egg distribution on egg survival was tested separately, egg survival was only lower when HR was induced by singly laid eggs (GLM, χ² = 4.07, df = 1, *P* = 0.04, Fig. 4B) and not by a clutch of 10 eggs (GLM, χ² = 1.99, df = 1, *P* = 0.16, Fig. 4B).

**Figure 5:**
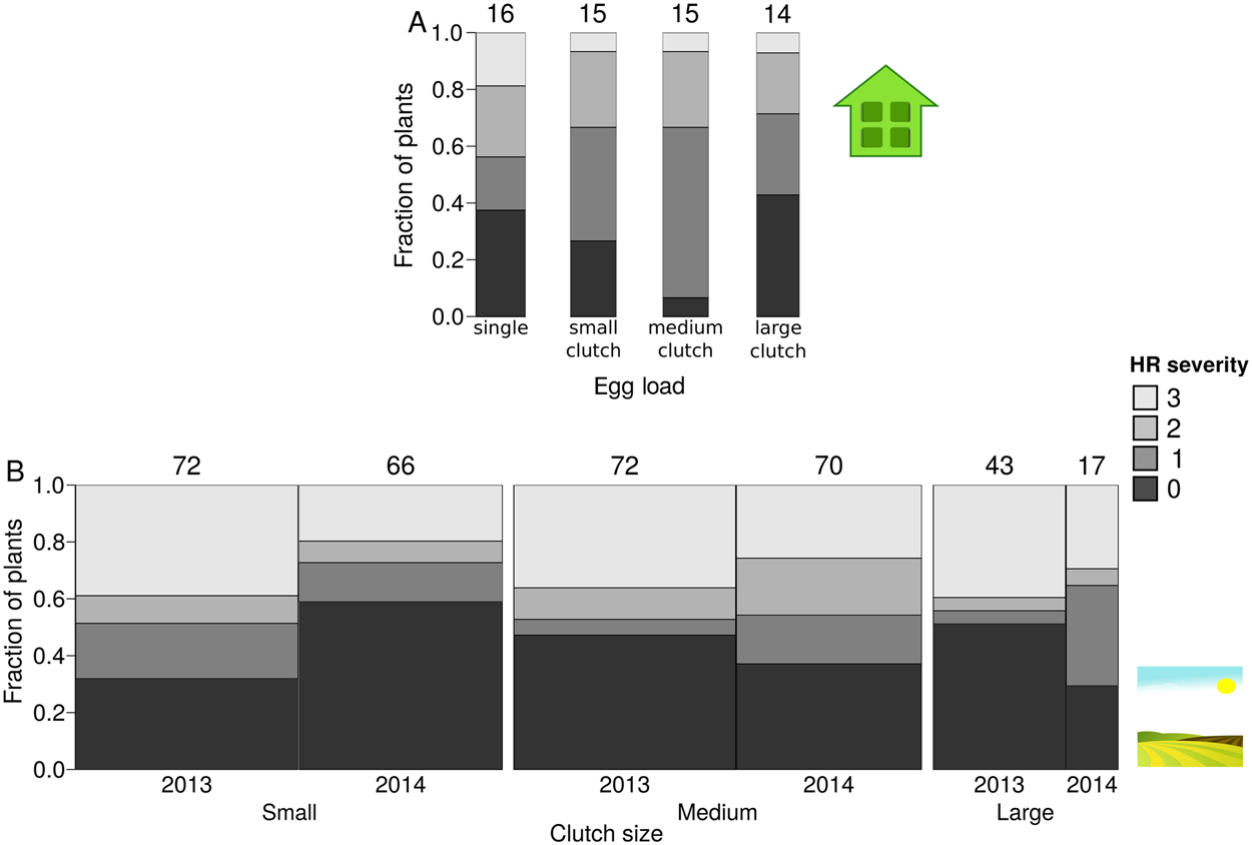
Determination of HR severity induced by different numbers of *P*. *brassicae* eggs distributed singly or clustered in different clutch size on *B*. *nigra* plants. Mean fractions of plants expressing different degrees of HR severity are given. Each shade of grey represents a different HR severity, and the numbers above each column show the number of plants tested (also represented by the width of the bars). (**A**) Under greenhouse conditions with single *P*. *brassicae* eggs and *P*. *brassicae* egg clusters of different size (small: 10, medium: 50 and large: 90 eggs) in categories as stated on the X-axis. (**B**) Under field conditions with the following categories of egg clutch size: Small: ≥6 < 50 eggs, Medium: ≥50 < 100 eggs and Large: ≥100 eggs.

Moreover, both in the greenhouse and in the field, the number of eggs laid per plant did not affect whether HR was expressed or not. Under greenhouse conditions no significant differences were found between plants with varying numbers of eggs (a single egg, groups of 10, ca. 50, ca. 90) in their probability of HR expression (GLM, χ² = 0.001, df = 1, *P* = 0.98) or the severity of the HR expressed (GLM, χ² = 0.313, df = 1, *P* = 0.58, Fig. 5A). Under field conditions no significant differences in probability of HR expression were found between plants induced by eggs laid in different clutch sizes (GLM, χ² = 0.003, df = 1, *P* = 0.96). For the severity of expressed HR no significant differences were found either (GLM, χ² = 3.45, df = 1, *P* = 0.06, Fig. 5B). However, in the field one genotype (SF29) showed a significantly higher probability to express HR in response to medium-sized egg clutches compared with small ones (Supplementary Figure S1).

To conclude, the results suggest that HR is an effective defence strategy against single eggs, rather than clustered eggs and that plants do not increase expression or severity of HR with an increasing number of eggs laid.

### Effects of desiccation on egg survival

Since humidity conditions at necrotic leaf tissue are expected to differ from those at live, transpiring tissue, we tested how survival of *Pieris* eggs depends (i) on humidity and (ii) on whether eggs were laid singly or in a clutch (with five eggs).

**Figure 6:**
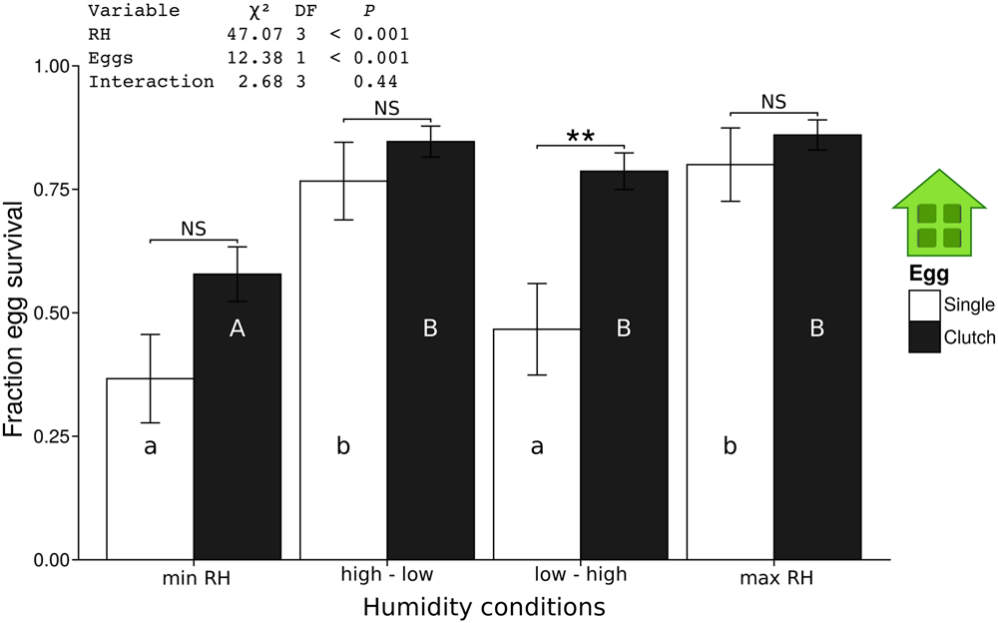
Effect of various humidity conditions on survival of *P*. *brassicae* eggs (mean fraction ± SE) until larval hatching. For the water/humidity treatment: compare Fig. S8. Different lowercase letters indicate significant differences in survival (*P* < 0.05, LHT post hoc test) of single eggs kept at different humidity conditions, while capital letters indicate significant differences in survival rates (*P* < 0.05, LHT post hoc test) of five eggs (clutch) kept at different humidity conditions. Asterisks above bars indicate whether or not survival rates of eggs differ between single and clustered eggs kept at a particular humidity treatment (LHT post hoc test). NS = Not significant, **P < 0.01, GLM. N per treatment = 30.

Overall, eggs in groups of five showed higher survival rates than single eggs (GLM, χ² = 12.38, df = 1, *P* < 0.001). The humidity conditions applied here significantly affected egg survival (GLM, χ² = 47.07, df = 3, *P* < 0.001, Fig. 6), while the interaction between humidity and egg group/singly laid egg did not (GLM, χ² = 2.68, df = 3, *P* = 0.44).

When considering egg survival rates at distinct humidity conditions, however, eggs in clutches of five showed a significantly higher survival rate for the treatment ‘low – high RH’ (GLM, χ² = 10.45, df = 1, *P* = 0.001), but not for the ‘min RH’ treatment (GLM, χ² = 3.02, df = 1, *P* = 0.08), ‘high – low RH’ (GLM, χ² = 0.99, df = 1, *P* = 0.32) or max RH treatment (GLM, χ² = 0.60, df = 1, *P* = 0.44, Fig. 6). The min RH treatment resulted in a significantly lower survival of eggs laid in a clutch compared to all other water treatments (Fig. 6). On the other hand, survival of singly laid eggs was significantly lower when no water was added in the beginning of the experiment (min RH and low – high RH treatments, Fig. 6). The weight loss/gain of eggs was different between single and clustered eggs. Single eggs lost or gained weight faster than the clustered eggs (Supplementary Figure S2). However, these differences were only significant for one time period in the high to low humidity treatment (between 51.625 h and 70.25 h after starting, Tukey HSD, *P* = 0.003, Fig. S2) and at two time periods in the low to high treatment (between 3.125 h and 22.875 h after starting, Tukey HSD, *P* = 0.001 and between 51.625 h and 70.25 h after starting Tukey HSD, *P* < 0.001, Supplementary Figure S2). The weight fluctuations are probably due to the loss and uptake of water and/or respiration. The stronger weight fluctuation in single eggs exposed to changing humidity conditions might explain their lower survival rates.

### Effects of abiotic factors

None of the weather factors (sum daily rainfall, relative humidity, mean daily temperature and mean total daily radiation) was correlated with the fraction of plants expressing HR in both experimental field seasons. None of the parameters had a significant impact on the fraction of egg clutches inducing HR (GLM, for (a) χ² = 0.84, df = 1, *P* = 0.36, (b) χ² < 0.001, df = 1, *P* = 0.98, (c) χ² = 0.01, df = 1, *P* = 0.91 and (d) χ² = 0.82, df = 1, *P* = 0.37, Supplementary Figure S3). Egg survival was tested under the above-mentioned weather factors, the expression of HR and the interaction between the weather factor and HR expression. Mean sum of the daily rainfall did significantly influence egg survival (GLM, χ² = 4.95, df = 1, *P* = 0.03, χ² = 0.09, Supplementary Figure S4) Interestingly, survival rates were highest when rainfall was moderate, indicating again that higher air humidity benefits egg survival, while highest rainfall brought the egg survival back to the same level as low rainfall. Neither HR expression nor the interaction of both changed egg survival (GLM, df = 1, *P* = 0.77, χ² = 0.28, df = 1, *P* = 0.60, Supplementary Figure S4). None of the other factors significantly affected egg survival (Supplementary information).

## Discussion

Our study reveals that plant genetic background influenced the likelihood and severity of HR induced by *P*. *brassicae* eggs under natural conditions. Interestingly, this variation in an egg-killing plant defence trait was not effective against a butterfly that deposits eggs in clusters. We show that the larval hatching rate from *P*. *brassicae* eggs laid in clutches of different sizes on *B*. *nigra* plants expressing HR - like necrosis is not affected by clutch size, neither under greenhouse nor under natural conditions. Only when butterflies were forced to lay single eggs, egg survival was lower when leaf necrosis was induced. Our data show that singly laid eggs suffer a stronger decrease in survival under low humidity conditions than eggs deposited in groups, which could explain the differences in the detrimental effect of HR – like necrosis on single and clustered eggs.

We hypothesize that formation of HR necrosis evolved as a defensive trait against lepidopteran specialists of brassicaceous plants. Two tested generalist moth species, *Spodoptera exigua* and *Mamestra brassicae* did not induce HR necrosis in *B*. *nigra* ^40–42^. A cue common to egg deposition by *Pierid* species might trigger HR. *Pieris brassicae* is the only *Pierid* species in The Netherlands that clusters eggs on brassicaceous plants^47^. This oviposition behaviour might render HR-like necrosis useless as a defence against eggs in this specific case.

An attenuating effect of egg clustering on the influence of an induced plant defensive response was also shown for the interaction between *Viburnum* plant and eggs of the viburnum leaf beetle (*Pyrrhalta viburni*): the beetle females prefer to oviposit on egg-infested *Viburnum* twigs by inserting their eggs into a cavity which the gravid female gnaws into the twig^21, 48^. Egg deposition induces the twig to grow wound tissue which squeezes the eggs. When eggs are aggregated in higher numbers, this egg-induced response is mitigated^21^.

Even though egg clustering seems very effective to counteract inducible egg-killing defences, it may increase the risk of parasitism and predation. Egg clusters might be easier detectable by visually orienting predators like birds than singly laid eggs. Furthermore, insect egg clusters might also be attractive targets for small egg parasitoids like *Trichogramma* spp. as they can easily parasitise many eggs after having encountered a clutch^49, 50^. Besides possible predation or parasitism risks, cannibalism is common in gregarious species. Upon hatching, *P*. *brassicae* caterpillars both eat their own egg shell and sometimes cannibalise the eggs of their siblings^51^ (E. Griese, pers. obs.). Finally, egg clustering may lead to competition for food among the caterpillars if the resources are limited. Some or all of these factors might have led to the situation where most species belonging to the Pierini such as *P*. *rapae* lay single eggs despite the fact that egg clustering can protect eggs from desiccation due to HR-like necrosis.

From the plant’s perspective, successful egg-killing defences reduce the chance of larval feeding damage. Gregariously feeding *P*. *brassicae* larvae can completely defoliate a *B*. *nigra* plant and cause irreversible damage to flowers (E. Griese & D. Lucas-Barbosa pers. obs.). In a previous study, it was shown for the solitary *P*. *rapae* that the percentage of eggs inducing HR is not dependent on the number of singly laid eggs on the plant, ranging from 2 to 9. However, formation of strong HR resulted in significantly reduced survival of the singly laid *P*. *rapae* eggs^14^. For *Heliconius subflexa*, the survival of singly laid eggs on *Physalis* sp. plants was lower when HR and/or neoplasm formation were expressed^12^. These examples corroborate our observation that singly deposited eggs of butterflies are affected by egg-killing plant responses.

Because plants will suffer more feeding damage from larvae hatching from egg clutches than from a few singly laid eggs, plants likely benefit from expressing a stronger HR - like necrosis against a higher number of eggs laid in a cluster. However, we did not find a correlation between the number of eggs laid (in a single clutch) and the probability or strength of HR formation. Moreover, the hatching success of *P*. *brassicae* larvae was not reduced by HR. Only when *P*. *brassicae* butterflies were forced to lay eggs singly onto the plant, the expression of HR reduced the hatching success. Our data indicate that *P*. *brassicae* can benefit from laying eggs in clutches. So far, it has been suggested that egg clustering increases the reproductive success of female butterflies^17^, especially when host plants are scarce. Hence, egg clustering may be beneficial for *P*. *brassicae* for two reasons: on the one hand it may be a cost-saving behaviour with respect to the energy invested in searching for oviposition sites, and on the other hand it obviously counteracts the negative effects of an egg-inducible direct plant defence trait, i.e. HR-like leaf necrosis.

The present study reveals that plant accessions differ in HR inducibility (likelihood of expressing HR) and the severity of HR expressed in response to insect egg deposition. Previous studies have shown genetic variation in plant defence traits only for defences against feeding herbivores (e.g. ref. 52), among them also laboratory and field studies of *Brassica* species investigating defence traits against feeding *Pieris* larvae. For example, laboratory experiments revealed that differences in volatile blends of *B*. *oleracea* var. *alba* L. varieties induced by feeding *P*. *brassicae* caterpillars lead to differences in attraction of a larval parasitoid. The differential attractiveness of these plant varieties to larval parasitoids matched different parasitism rates of larvae feeding on different varieties in the field; furthermore, herbivore preference and performance, as well as insect communities living on the plants varied among these plant varieties^53–55^. These results are in line with our findings, confirming that different genotypes of a *Brassica* species differ in their feeding- and oviposition-inducibility of anti-herbivore defence traits under field conditions.

Our data further show variation among individual plants in HR expression. Some plant individuals exhibited resistance to eggs and expressed an HR-like response, whereas others did not. It is likely that this variation is caused by a high variation in the responsiveness of a resistance (R) gene in *B*. *nigra* plants recognising specific cues of *Pieris* eggs. R-genes targeting phytopathogens are among the most polymorphic loci in plant genomes^56, 57^. Whether this is similar for R-gene(s) involved in HR against *Pieris* eggs is an open question. Studies of plant-pathogen co-evolution often report one type of interaction, the gene-for-gene interaction, whereby plants generate R products that recognise cues from the specific attacker^58^. Although the plant genotypes used in our study originated from a self-fertilisation event (produced by E.H. Poelman) (or crossing of two self-fertilised plants of the same genotype, produced by E.H. Poelman and E. Griese), they are not homozygous which may explain the observed variation in HR expression between individual plants of each accession.

Our data suggest that laying eggs in clusters reduces their susceptibility to HR-like necrosis. Formation of necrotic leaf tissue might lower the humidity around the egg clutch because of reduced plant transpiration at this site. Shapiro and DeVay^10^ suggest that HR kills eggs by reducing humidity at the interface between plant surface and eggs, thus resulting in detachment of eggs from the leaf or in egg desiccation. Water loss of eggs is determined by various factors. In addition to atmospheric humidity, transpiration of the plant tissue and other egg-related factors like the chemical composition of the eggshell, the serosa, size of the egg surface as well as the exocrine secretion attached to the eggshell may play a role in preventing egg desiccation^59–61^. Especially the formation of the serosa has been shown to drastically increase drought resistance in eggs of several insect species, including beetles and mosquito species^62–64^. Whether the serosa plays a similar role in butterfly eggs remains to be investigated. In addition to the mentioned parameters, aggregation of eggs could prevent desiccation under low humidity conditions, as was shown for *Chlosyne lacinia* butterflies feeding on the common sunflower *Helianthus annuus* ^51^. Their egg clutches can vary in size and the number of layers. A higher number of egg layers and larger clutch size improved the hatching success under low humidity conditions^51^. In the present study, we show that clustered *P*. *brassicae* eggs can recover better from low humidity conditions when transferred to high humidity than singly laid eggs; the survival rate of clustered eggs was higher under these conditions. This indicates that the strong, negative effects of HR-related necrosis on especially the singly laid eggs might also be due to reduced humidity at the oviposition site. Thus, by implication, egg aggregation can protect eggs against a detrimental HR-associated effect, i.e. low humidity at the oviposition site.

Unlike under field conditions, the different accessions did neither differ in the likelihood of expressing HR nor in the strength of HR expression when placed under greenhouse conditions. Furthermore, the proportion of plants that show HR as well as the average HR severity were generally lower. These results suggest that abiotic factors present in the field but not in the greenhouse (e.g. UV-light or higher temperature and humidity fluctuations), affect HR expression. However, in our field experiment, none of the particular abiotic factors that we determined affected the expression of HR in *B*. *nigra* in neither of the two seasons. Nevertheless, some plant accessions might have been more affected by changes in abiotic conditions in the field than others, which would explain the more similar HR expression of the accessions in the greenhouse. In contrast to our experimental field data, Petzold-Maxwell *et al*.^12^ showed that temperature was positively correlated with the likelihood of the expression of HR and neoplasm formation induced by eggs of the moth *Heliothis subflexa* in *Physalis angulata* plants in a field experiment; the temperatures measured in this study were higher than the highest temperature measured in our field study (ca. 21 °C). Furthermore, a previous study on *B*. *nigra* and *P*. *rapae* in California, U.S.A., indicated that HR necrosis was more pronounced at high temperatures (above 30 °C)^10^. The range of changes of abiotic factors as well as very high temperature and low humidity may affect the expression of HR.

Our study revealed that aggregating eggs is a strategy for *P*. *brassicae* to avoid negative effects of HR as a direct egg-killing response. However, there must be some fitness costs associated with egg clustering, because other common *Pieris* species (e.g. *P*. *rapae* and *P*. *napi)* lay single eggs^65^. Costs of egg clustering could be associated with increased larval competition on the same food source and/or increased egg and larval predation or parasitism risks. Future studies need to elucidate the ecological consequences that *B*. *nigra* plant genotypes and individuals face when not expressing HR-like necrosis in response to *P*. *brassicae* eggs.

## Methods

### Plants and insects

*Brassica nigra* L. plants were grown either in a greenhouse (18 ± 5 °C, 50–70% RH, L16: D8) or, for field experiments, first grown in the greenhouse for ca. one week, and then placed in an outdoor area protected from wind and rain for two to three weeks. For the experiments in 2013, we used seeds of two self-fertilised plants (SF19 and SF48) that had been collected in 2009 from a *B*. *nigra* population near the River Rhine in Wageningen, The Netherlands (N51.96, E05.68). In 2014, seeds produced from plants of the original self-fertilised plants of the same accessions- and of two additional accessions were used (SF19, SF25, SF29 and SF48).

*Pieris brassicae* L. (Lepidoptera: Pieridae) was reared on Brussels sprouts plants (*B*. *oleracea* var. *gemmifera* cv. Cyrus) in a climate room (21 ± 1 °C, 50–70% RH, L16: D8). Freshly emerged virgin female and male *P*. *brassicae* butterflies were obtained from the rearing and kept isolated. Two days after emergence they were allowed to mate, and two days later the mated female butterflies were used for egg deposition in the experiments.

### Experimental conditions

Common garden experiments under field conditions. Research questions 1, 3 and 5 were tested in a common garden setup, conducted in two consecutive years (2013 and 2014). In both years, young greenhouse-grown seedlings (three weeks old) were planted into the field in three different periods, two weeks apart from each other, starting mid-May and ending end of July. The seedlings were planted in fields of the experimental farm of Wageningen University (Unifarm), The Netherlands. Several *B*. *nigra* accessions were planted in the field. In 2013, SF19 and SF48 were grown, and in 2014 we planted SF19, SF25, SF29 and SF48. In total 192 plants in 2013 and 240 in 2014 were infested with *P*. *brassicae* eggs. The plants were organised into plots namely in 12 rows of four plots each in 2013 and in 15 rows of four plots each in 2014. Each plot contained four egg-infested plants with three metre distance between the plots. A mixture of *Lolium* and *Poa* grasses was sown in between the plots. The egg-infested plants were grown about two metre apart from each other within a plot. Half of the plants were covered with a fine mesh protecting eggs from predators and parasitoids. Plants were infested with eggs when they were four to five weeks old. One to two weeks after planting, each plant was exposed to a mated female which was kept on a plant until egg deposition (between 0.5 to 24 h) by covering it with a gauze net. After deposition of one egg cluster (additional clusters were gently removed), the leaf with the egg cluster was photographed for documentation and later counting of the eggs per cluster.

Greenhouse experiments. Research questions 1 to 4 were also addressed under greenhouse conditions (22 ± 2 °C, 50–70% RH, L16: D8).

### Egg survival

The survival rate of eggs was calculated based on the total number of eggs and the number of caterpillars that hatched (hatched caterpillars/total number of eggs). The number of caterpillars was counted just after hatching by visual observation or by taking a picture and counting eggs and caterpillars with the help of the computer programmes ImageJ as described by Schneider *et al*.^66^ and Gimp (see http://www.gimp.org/).

### Determination of HR-like necrosis

**Figure 7:**
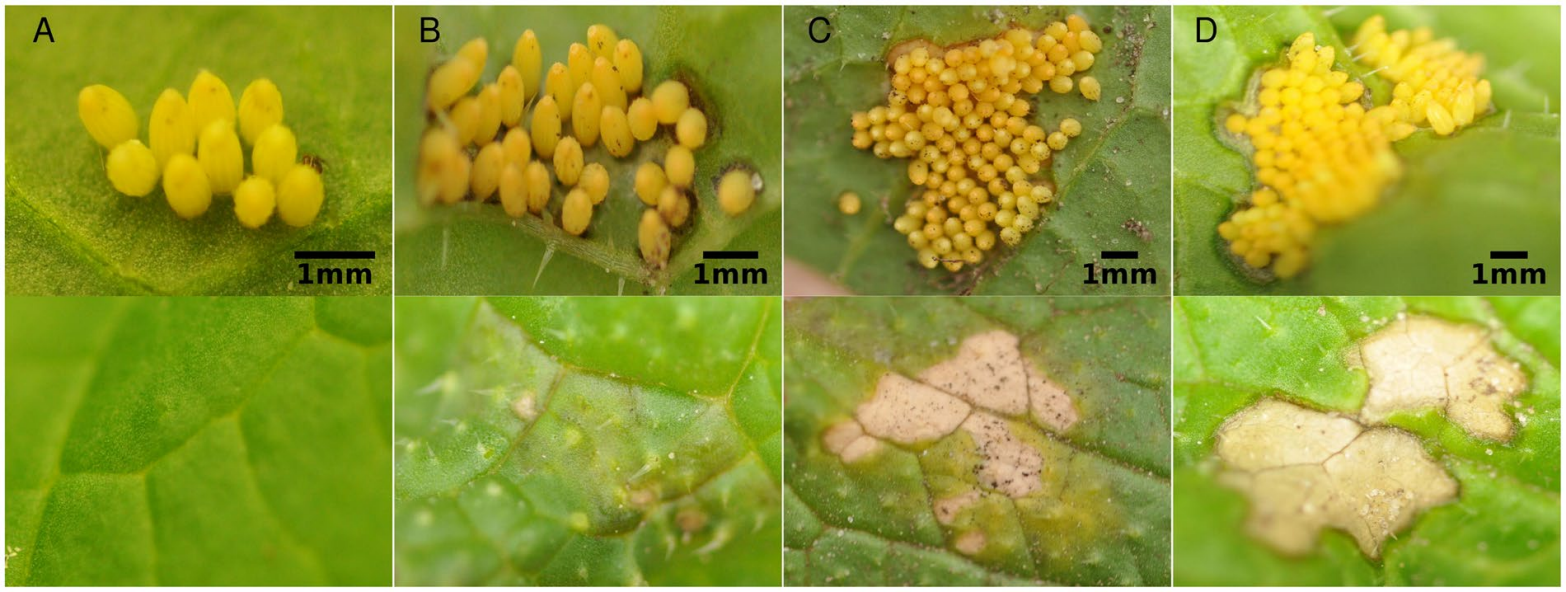
Different severity levels of HR - like necrosis induced by egg clutches of *P*. *brassicae* laid onto *B*. *nigra* plants under field conditions. The upper row shows the abaxial side of a leaf with eggs, the lower row shows the adaxial side of a leaf. (**A**) No HR - like necrosis induced (no HR = 0), (**B**) HR severity 1: necrosis can be observed below eggs, but hardly on the other side of the leaf, (**C**) HR severity 2: necrosis has spread and can be clearly observed on both sides of the leaf, the edges of the reaction are diffuse. (**D**) HR severity 3: necrosis is strongly expressed also around the egg clutch with sharp edges. All clutches shown are the same age (four days after egg deposition).

All plants in the field and greenhouse were visually checked for HR symptoms three to four days after egg deposition (Fig. 7). The plants were categorised into ‘HR-‘ (no necrotic zone observed, Fig. 7A) or ‘HR+ ’ (with necrotic zone, Fig. 7A–D). When HR was observed, its severity was quantified by categorising it into three different classes: necrosis visible underneath the eggs (HR severity ‘1’) (Fig. 7B), necrosis also visible on the other side of the leaf (HR severity ‘2’) (Fig. 7C), and necrosis visible around the eggs and the other side of leaf with clear edges (HR severity ‘3’) (Fig. 7D). Four days after egg deposition, the HR is fully expressed and does not change any more.

### Effect of plant individual and genotype on HR expression

A four-week-old *B*. *nigra* plant from either the SF19 or SF48 accession was infested with eggs in the greenhouse as described above. A female butterfly was placed on a leaf for oviposition. As soon as it had laid five eggs, the butterfly was removed from the cage and a new female was introduced. After a plant had received eggs from eight to ten female individuals (five eggs per female), it was replaced by a new one and offered to the same set of butterflies. Hence, in total each plant received 40 to 50 eggs on a single leaf, a procedure which was repeated for eight to ten plants (half of them being SF19, half SF48) using the same set of females (Supplementary Figure S5). In total, four sets of eight to ten plants, each with eggs from a different set of eight to ten butterflies were prepared. The youngest leaf large enough to sustain all eggs (usually 3^rd^ or 4^th^ leaf from the top of the plant) was infested with eggs, and infestation took less than one hour per plant. The sequence of butterflies used for oviposition was randomised for each plant. We recorded HR-like symptoms and their severity induced by each egg cluster for a period of four days after egg deposition. Egg survival was recorded as well (as described above). Thus, these parameters were assessed for each plant individual.

### Effects of egg clutch size and egg clustering on HR expression and egg survival

To investigate whether the number of eggs laid affects HR severity under greenhouse conditions, *P*. *brassicae* butterflies were allowed to deposit eggs in three different clutch sizes on plants from two *B*. *nigra* accessions (SF19 and SF48). Four-week-old *B*. *nigra* plants were placed in a cage (35 × 60 × 40 cm) together with one *P*. *brassicae* female to obtain either 1, 10, 50 or 90 eggs per plant. Additional eggs were gently removed right after discovery using a soft brush. Fourteen to sixteen plants per treatment were infested.

To investigate further whether the type of egg distribution (singly laid eggs *versus* clustered eggs) impacts on the effects of HR on egg survival when plants are deposited with the same number of eggs, we conducted a further experiment in which *B*. *nigra* plants were always infested with 10 *P*. *brassicae* eggs. The eggs were either laid as a clutch or singly. The 10 singly laid eggs were at least 5 mm apart but still on the same leaf. For each egg distribution mode we used 32 plants. However, one female which was used for both egg distributions laid unfertilised eggs, and one egg-deposited leaf of a plant used for the single egg distribution broke off during the experiment, thus reducing the number of plants to 30 (for singly laid eggs) and 31 (for clustered eggs).

Under field conditions, we recorded HR-like symptoms and their severity induced by each egg cluster for a period of four days after egg deposition. We correlated the different egg clutch sizes with formation of HR and egg survival.

### Effect of abiotic factors on HR expression and egg survival

#### Microclimate

Egg-induced formation of necrotic leaf tissue is likely to be associated with changes in humidity at the interface between plant surface and eggs. To investigate whether differences in humidity and changes in humidity affect single and aggregated eggs differentially, a laboratory experiment was conducted in which freshly laid eggs were kept in a climate cell under controlled temperature and light conditions (24 ± 0.5 °C, L16: D8) but different humidity conditions (Supplementary Figure S6). First, a filter paper disc was laid into a Petri dish (9.4 cm diameter), and lids of small Petri dishes (5.8 cm diameter) were placed onto the filter paper discs. Eggs were carefully removed from *B*. *oleracea* var. *gemmifera* plants and transferred singly or in a group of five into the cap of an Eppendorf tube (1.5 ml). The eggs had been laid maximally two hours before the experiment. Five caps with eggs were placed onto each small Petri dish lid. Then the large Petri dishes (9.4 cm diameter) were closed. The set up was chosen to avoid direct contact of eggs with the water. The following humidity conditions were generated: (i) The filter paper was completely soaked with 2 ml water after placing the eggs into the dishes. We assumed that the relative humidity was constantly kept at nearly 100% under the closed lid during the whole egg-development period (4.5 days) (max RH treatment). (ii) A dry environment of the eggs (mimicking plant cell necrosis) was reached by not adding water (min RH treatment). (iii) The effect of change in humidity on egg survival was tested by adding 1 ml water at the beginning of the experiment, and then allowing complete evaporation (humidity changes from high to low (referred to as high-low RH). (iv) The reverse treatment (humidity changes from low to high) was created by adding 1 ml water only 2.5 days after placing the eggs into the setup (referred to as low-high RH). The hatching success was measured after four to five days.

#### Macroclimate

Weather data for the periods in which the common garden experiments were conducted (see above) were obtained from the Veenkampen weather station of Wageningen University, around 3.25 km west of the field site. Data on the daily mean temperature (°C), the sum of all net radiation per day (W/m²), mean relative humidity (%) and the sum of daily rainfall were collected for all days of the experiment during the field season. Their values over the four days following egg deposition were used to calculate a mean ± SE value. The mean value was checked for correlation with expression of HR and its severity. The mean value was also correlated with the mean value of egg survival for the period of time.

### Statistical analysis

Generalised linear models (GLM) were used as the main tool for statistical analysis for all experiments. Egg survival and HR expression as response variable were analysed using a binominal distribution. The HR severity as response variable was analysed using a Poisson distribution. To compare different factor levels within a model pairwise Mann-Whitney – U tests were conducted. Alternatively, a Tukey HSD test was conducted if applicable. All tests were conducted using the statistic platform R 3.2.5^67^.

### Data Availability

The datasets generated during and/or analysed during the current study are available from the corresponding author on reasonable request.

## Electronic supplementary material

Supplemetary information and figures
