# Supplementary material for: Plant response to butterfly eggs: inducibility, severity and success of egg-killing leaf necrosis depends on plant genotype and egg clustering
Source: Sci Rep. 2017 Aug 4;7:7316. doi: 10.1038/s41598-017-06704-z (PMC5544688; doi:10.1038/s41598-017-06704-z)
Supplement: Supplementary file 1 — Supplemetary information and figures [file 41598_2017_6704_MOESM1_ESM.pdf]

Scientific Reports Supporting Information

Plant response to butterfly eggs: inducibility, severity and success of egg-killing leaf necrosis depends on plant genotype and egg clustering

Eddie Griese, Marcel Dicke, Monika Hilker and Nina E. Fatouros

The following Supporting Information is available for this article:

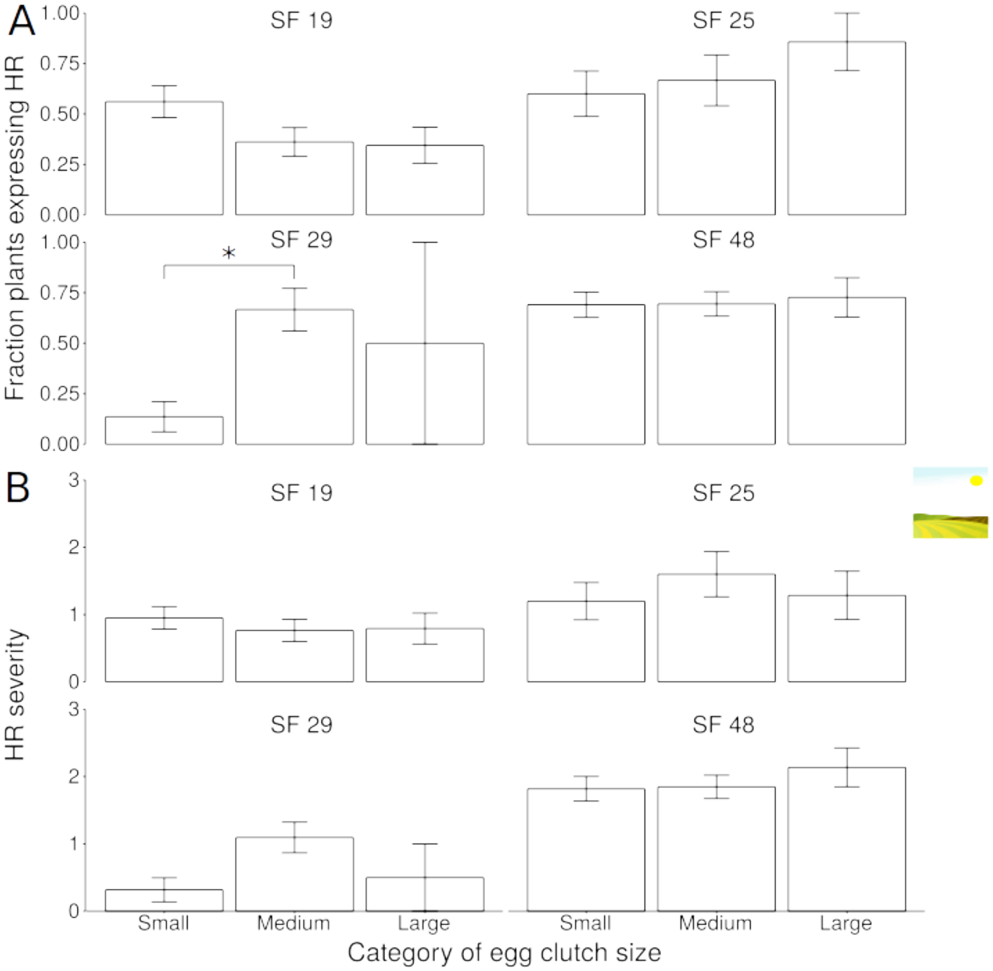

Fig. S1 Relationship between egg clutch size (in categories, Small: 6 – 50 eggs, Medium: 51 – 100 eggs, and Large: more than 100 eggs) and (A) the fraction of plants expressing HR, and (B) the fraction of plants showing a particular HR severity, as recorded in field experiments. All four plant accessions are separately depicted, and only significant differences are shown to keep the graph simple (*: $P < 0.05$).

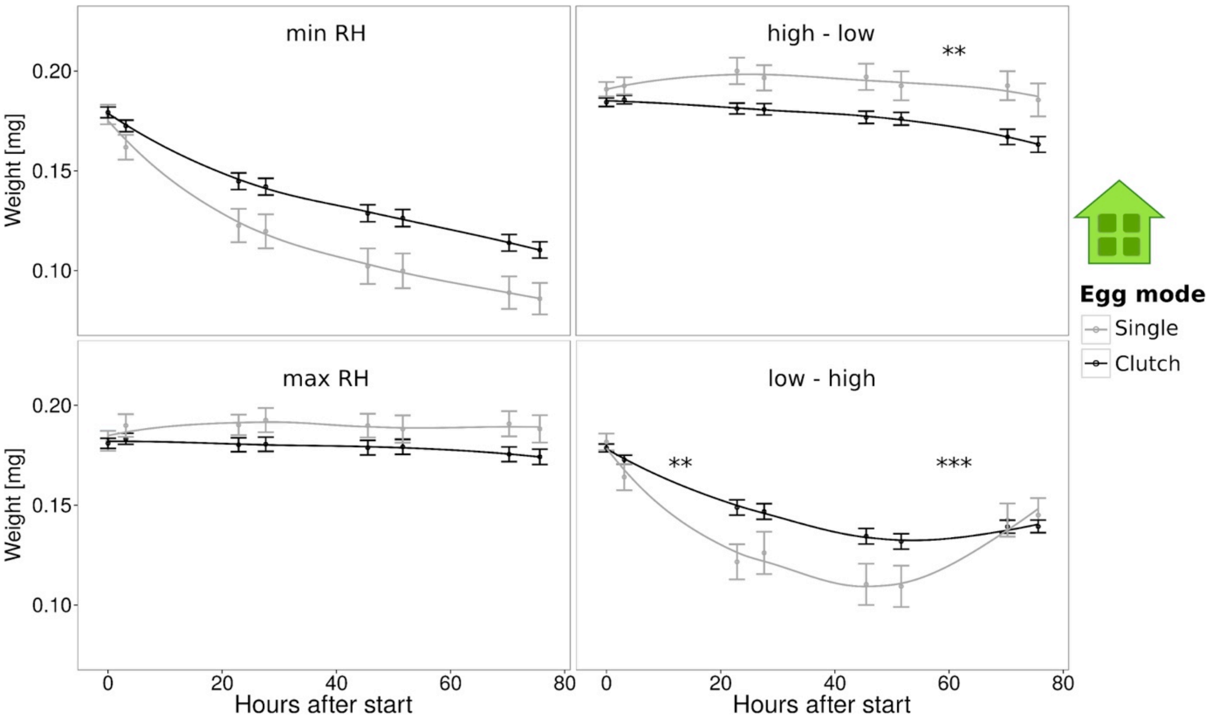

Fig. S2 Weight gain or loss of single eggs or egg clutches exposed to various humidity conditions (compare Fig. S4 for humidity conditions). Data points are shown as means (± SE). Asterisks between two measuring points indicate significant differences in weight change between single and clustered eggs. **: $P < 0.01$, ***: $P < 0.001$. For greater clarity only significant results are marked.

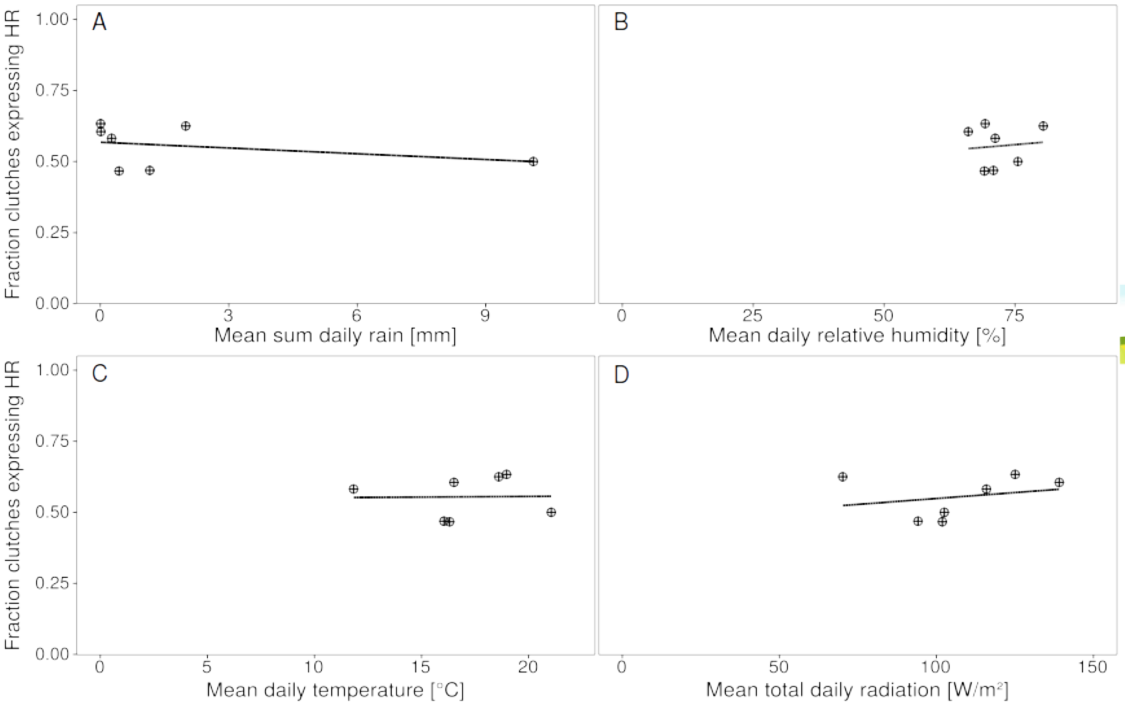

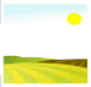

Fig. S3 Relationship between the fraction of egg clutches inducing HR in the field and abiotic conditions during 2013 and 2014. (A) Mean sum of daily rainfall. (B) Mean daily relative humidity. (C) Mean daily temperature. D) Mean daily total radiation.

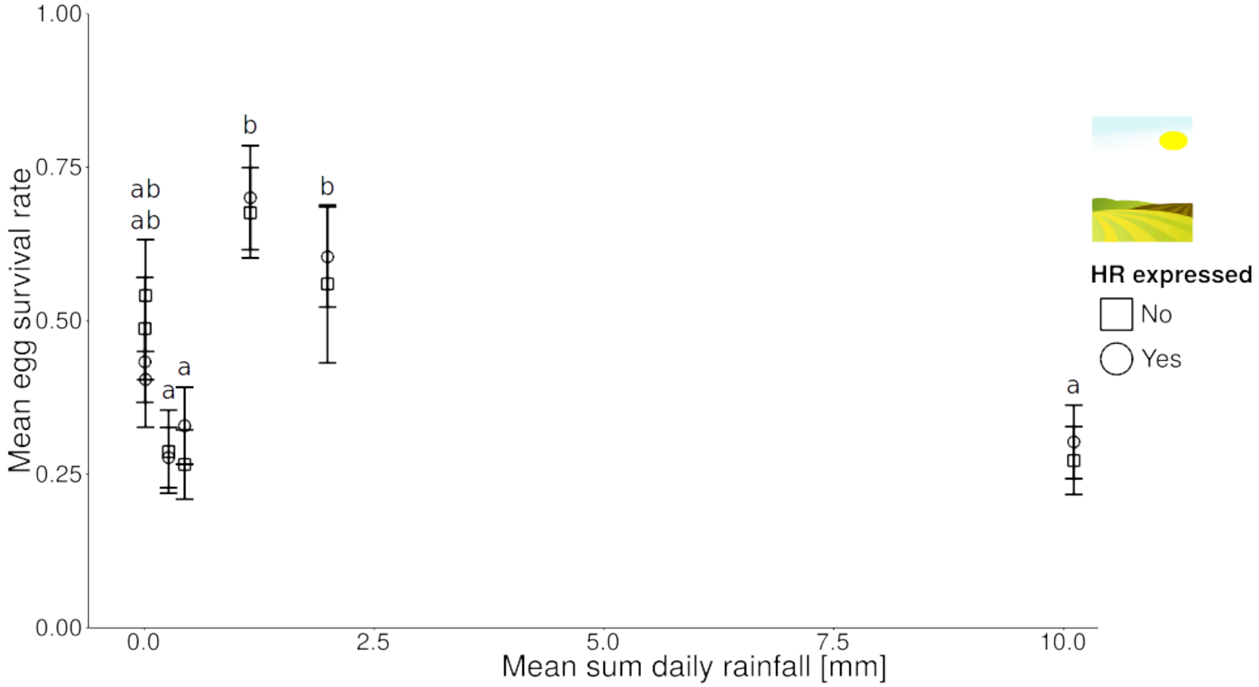

Fig. S4 Egg survival rate in the field under different rainfall conditions. The sum of daily rainfall did significantly influence egg survival under field conditions (different letters indicate $P = <0.05$, pairwise MWU test).

Abiotic factors

None of the other tested abiotic factors affected egg survival, namely relative humidity (GLM, $\chi^2 = 0.39$, df = 1, $P = 0.53$, $\chi^2 = 0.14$, df = 1, $P = 0.71$, $\chi^2 = 1.92$, df = 1, $P = 0.17$), mean daily temperature (GLM, $\chi^2 = 0.57$, df = 1, $P = 0.45$, $\chi^2 = 0.17$, df = 1, $P = 0.68$, $\chi^2 = 0.35$, df = 1, $P = 0.56$) and mean total daily radiation (GLM, $\chi^2 = 3.05$, df = 1, $P = 0.08$, $\chi^2 = 0.16$, df = 1, $P = 0.69$, $\chi^2 = 2.24$, df = 1, $P = 0.13$).

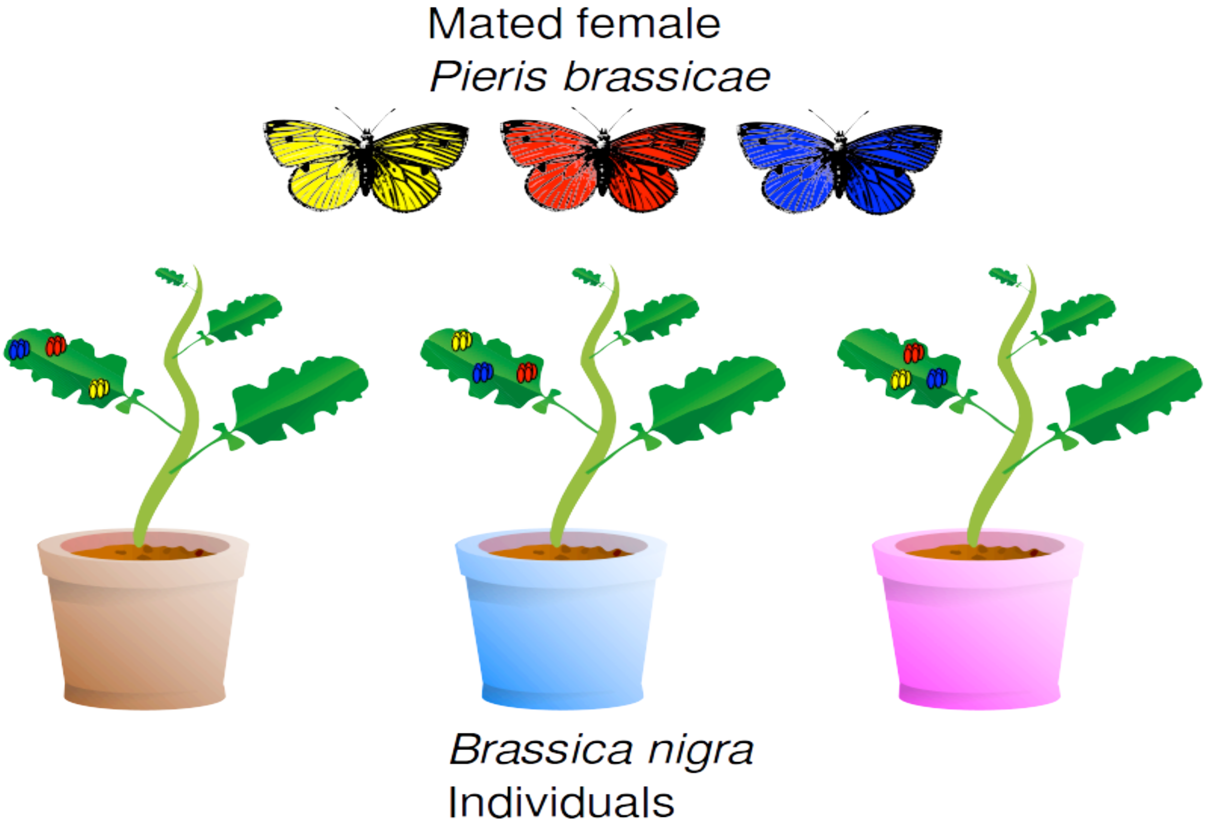

Fig. S5 Schematic outline of the experiment on the impact of a plant individual on formation of HR – like necrosis. Each butterfly individual was allowed to lay a clutch of five eggs (eggs of the same colour originate from the same butterfly) per plant (represented through differently coloured pots). While only three butterflies and plants are shown here, for the experiment eight to ten plants and butterflies were used.

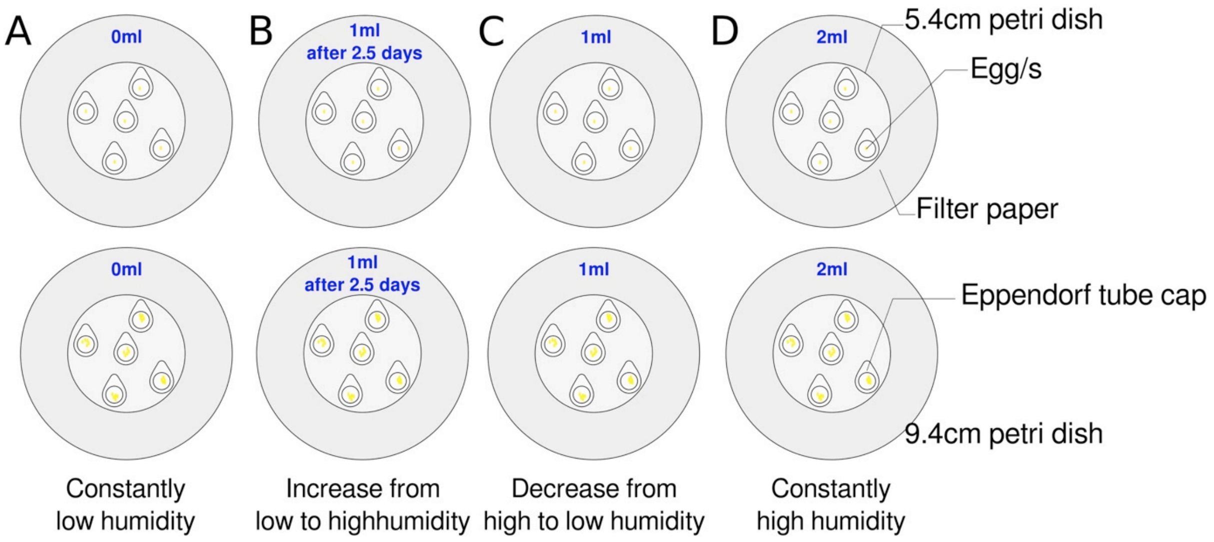

Fig. S6 Experimental setup for investigating the impact of various humidity conditions on egg survival. Eggs were placed into Eppendorf caps for easy weighing and also protecting them from direct contact with the water on the filter paper. (A) 0 ml water was added to the filter paper and the dish was kept closed for 4.5 days ("min RH"). (B) 1 ml was added to the filter paper, but only 2.5 days after placing the eggs in the dish; the dish was kept closed ("low –> high RH"). (C) 1 ml water was added, slow evaporation over time reduced the water content to 0 ml ("high –> low RH"). (D) 2 ml water were added; eggs were kept at humid conditions for 4.5 days. Larval hatching rates were recorded to determine egg survival rates.
